# Inhibition of miR-9-5p suppresses prostate cancer progress by targeting StarD13

**DOI:** 10.1186/s11658-019-0145-1

**Published:** 2019-03-08

**Authors:** Lin Chen, Weifeng Hu, Guohao Li, Yonglian Guo, Zhihua Wan, Jiajun Yu

**Affiliations:** Department of Urology, The Central Hospital of Wuhan, Tongji Medical College, Huazhong University of Science and Technology, No. 26 Shengli Street, Jiang’an District, Wuhan, 430014 China

**Keywords:** microRNA-9-5p, Prostate cancer, StarD13, Migration, Invasion

## Abstract

**Background:**

This study aims to investigate the effects of inhibiting microRNA-9-5p (miR-9-5p) on the expression of StAR-related lipid transfer domain containing 13 (StarD13) and the progress of prostate cancer.

**Methods:**

The mRNA expression levels of miR-9-5p and StarD13 were determined in several prostate cancer cell lines. We chose DU145 and PC-3 cells for further research. The CCK8 assay was used to measure the cell viability. The cell invasion and wound-healing assays were respectively applied to evaluate invasion and migration. The expression of E-cadherin (E-cad), N-cadherin (N-cad) and vimentin were measured via western blot. DU145 and PC-3 cells overexpressing StarD13 were generated to investigate the variation in proliferation, invasion and migration. A luciferase reporter assay was used to identify the target of miR-9-5p.

**Results:**

Our results show that miR-9-5p was highly expressed and StarD13 was suppressed in prostate cancer cells. MiR-9-5p inhibition repressed the cells’ viability, invasion and migration. It also increased the expression of E-cad and decreased that of N-cad and vimentin. StarD13 overexpression gave the same results as silencing of miR-9-5p: suppression of cell proliferation, invasion and migration. The bioinformatics analysis predicted StarD13 as a target gene of miR-9-5p. Quantitative RT-PCR, western blot analysis and the dual-luciferase reporter assay were employed to confirm the prediction.

**Conclusion:**

Our results show that miR-9-5p plays a powerful role in the growth, invasion, migration and epithelial–mesenchymal transition (EMT) of prostate cancer cells by regulating StarD13. A therapeutic agent inhibiting miR-9-5p could act as a tumor suppressor for prostate cancer.

## Background

Prostate cancer is the most common cancer in men with the third highest mortality in the United States, behind lung and bronchia cancer [1]. While the incidence and mortality rates for prostate cancer were significantly lower in Asian countries than in western ones [2], the morbidity and mortality of prostate cancer in Asia have steadily increased in recent years, showing a more rapid rate of growth than in the West [3]. Developing novel targets that regulate the progress of prostate cancer is thus an important research goal worldwide.

MicroRNAs (miRNAs) are a class of 22-nucleotide noncoding RNAs encoded by endogenous genes. They regulate gene expression levels by binding to the 3′-untranslated region (UTR) of target mRNAs. Recent studies showed that miRNAs can be used as diagnostic and prognostic biomarkers of prostate cancer [4], with miR-1271 [5], miR-1297 [6], miR-126 and 149 [7] positively identified as involved in the process.

In humans, miR-9 is transcribed from three independent genomic loci mapping to chromosomes 1q22 (MIR9–1), 5q14.3 (MIR9–2) and 15q26.1 (MIR9–3). Their primary transcripts ultimately give rise to the functionally mature miR-9-5p [8]. Accumulating evidence suggests that miR-9-5p prompts malignancy in acute myeloid leukemia cells, mainly by targeting p27 [9]. One well-known study proved that miR-9-5p has the ability to enhance cell proliferation and invasion in non-small cell lung cancer [10]. A previous study reported that miR-9 serves as an oncomiR in prostate cancer, promoting tumor progress and metastasis [11]. Thus, miR-9-5p is implicated in the regulation of cancer cell proliferation, invasion and migration. However, the precise role and underlying mechanisms of miR-9-5p regulation in prostate cancer remains unknown.

Epithelial–mesenchymal transition (EMT) is a process by which epithelial cells lose their polarity and are converted to a mesenchymal phenotype. It has been suggested as a pivotal step for cancer invasion and metastasis [12, 13]. Activation of EMT is related to aberrant expression of a variety of genes. It is commonly characterized by downregulation of E-cadherin (E-cad), which is a vital epithelial marker, accompanied by upregulation of N-cadherin (N-cad) and vimentin, which are crucial mesenchymal marker genes.

StAR-related lipid transfer domain containing 13 (StarD13), a GAP for Rho GTPases, has been confirmed as a tumor suppressor. It shows low expression in a number of tumors, including lung, renal, breast and colon tumors [14–16]. A previous study reported that the StarD13-correlated ceRNA network suppressed breast cancer migration, invasion and EMT [17]. As a target of several miRNAs, StarD13 plays a critical role in regulating tumor progression. For instance, miRNA-125b promotes the invasion and metastasis of gastric cancer cells by targeting StarD13 and NEU1 [18]. Importantly, it has been well documented that StarD13 is directly targeted by miR-9 in triple-negative breast cancer [19]. However, the regulatory relationship in prostate cancer remained to be elucidated.

In this study, we investigated the role of miR-9-5p in the development of prostate cancer. Bioinformatics analysis predicted that StarD13 is a target gene of miR-9-5p. Therefore, an miR-9-5p inhibitor was transfected into DU145 or PC-3 cells to evaluate the effects of via targeting StarD13 on the cells’ growth, invasion, migration and EMT. We aimed to show whether miR-9-5p and StarD13 might act as a novel therapeutic target for the treatment of prostate cancer.

## Materials and methods

### Cell culture and transfection

Human normal prostate epithelial cell line RWPE-1 was obtained from the Cell Bank of the Chinese Academy of Sciences and cultured in keratinocyte-SFM (K-SFM) medium. The human prostate cancer cell lines DU145, 22RV1 and PC-3 were purchased from the American Type Culture Collection (ATCC). DU145 cells were cultured in Eagle’s minimum essential medium (MEM) containing 10% fetal bovine serum (FBS) whie 22RV1 and PC-3 cells were incubated in RPMI 1640 medium. All cells were cultured in a 5% CO_2_ atmosphere at 37 °C.

An miR-9-5p inhibitor and its negative controls (NC) were synthesized by Shanghai Gene Pharma. Both were used at a final concentration of 50 nM. DU145 and PC-3 cells were transfected with the miR-9-5p inhibitor or miR-NC using Lipofectamine 2000 (Invitrogen) according to the manufacturer’s protocol.

The overexpression plasmid of StarD13 was purchased from Shanghai Gene Pharma. Then cells were transfected with the overexpression plasmid or empty vector using Lipofectamine 2000 (Invitrogen). Successful transfections were determined using quantitative RT-PCR or the western blot assay after incubation for 48 h.

### Quantitative RT-PCR

Total RNA was collected from cells using Trizol Reagents (Invitrogen) and quantified using a spectrophotometer (Bio-tek). Then a total of 1 μg total RNA was reverse transcribed into complementary DNA (cDNA) using a PrimeScript RT Reagent Kit (Perfect Real Time; TaKaRa Biotechnology). The cDNA was quantified via real-time quantitative PCR using a SYBR Green Master Mix Kit (TaKaRa Biotechnology). The sequence of miR-9-5p is: 3′-AGUAUGUCGAUCUAUUGGUUUCU-5′. The miR-9-5p expression was normalized to U6, and the expression of StarD13 was normalized to GAPDH. The data were analyzed using the 2^−ΔΔCt^ method [20]. The primers were:

- miR-9-5p, forward 5′-ACACTCCAGCTGGGAGTATGTCGATCTATTG-3′; reverse, 5′-TGGTGTCGTGGAGTCG-3′
- StarD13, forward 5′-CGAGGAGACAGAAATGGGTCA-3′; reverse 5′- TCCACTGCTTTCGCTGTGAAT-3′
- U6, forward 5′-CTCGCTTCGGCAGCACA-3′; reverse, 5′-AACGCTTCACGAATTTGCGT-3′
- GAPDH, forward 5′-GGAGCGAGATCCCTCCAAAAT-3′; reverse 5′-GGCTGTTGTCATACTTCTCATGG-3′

### CCK-8 assay

Cell proliferation was assessed with a CCK-8 kit (Dojindo Laboratories). DU145 cells or PC-3 cells were incubated in 96-well plates. After incubation for 24 h, the cells were left untreated treated (control) or were treated with the empty vector (negative control, NC) or with the miR-9-5p inhibitor for 12, 24, 48 or 72 h. CCK-8 was added into each well and incubated at 37 °C for 2 h. The absorbance was measured at 450 nm using a microplate reader (Bio-Rad 680).

### Wound-healing assay

After transfection with miR-9-5p inhibitor or NC, DU145 or PC-3 cell monolayers were scratched using a sterile 200 μl pipette tip and cultured with serum-free medium. The wound was observed and photographed with an Olympus invert microscope immediately and 24 h after scratching in 5 random (magnification, 5x) fields in each sample. The cell migration rate was calculated as:

$$ \mathrm{Mobility}\left(\%\right)=\left(24\hbox{-} \mathrm{h}\ \mathrm{scratch}\ \mathrm{distance}/\mathrm{initial}\ \mathrm{distance}\right)\times 100\% $$

### Cell invasion assay

The invasion ability of DU145 or PC-3 cells in the study groups was assessed using Matrigel-Coated Transwell Chambers (BD Bioscience). Cells were seeded into the upper chamber. After overnight incubation, cells in lower chamber were fixed with 4% paraformaldehyde, followed by staining with 0.1% crystal violet. They were counted under a microscope.

### Western blot analysis

After treatment, DU145 or PC-3 cells in different groups were harvested and lysed in ice-cold RIPA lysis buffer (Keygen Biotech) for 30 min. BCA assay (Beyotime Institute of Biotechnology) was carried out to measure the protein concentrations. Equal amounts of proteins (40 μg) were separated using 6–10% SDS-PAGE gels and then transferred to polyvinylidene difluoride (PVDF; Millipore) membranes. The membranes were then blocked with dried skimmed milk for 1.5 h at room temperature and immerged into the primary antibodies overnight at 4 °C (StarD13, sc-377,054, 1:1000, Santa Cruz Biotechnology; GAPDH, sc-32,233, 1:1000, Santa Cruz Biotechnology; E-cad, 3195S, 1:1000, Cell Signaling Technology; N-cad, 13116S, 1:1000, Cell Signaling Technology; anti-vimentin, 3300 T, 1:1000, Cell Signaling Technology). Then membranes were immerged into HRP-labeled Goat Anti-Mouse IgG (A0216, 1:1000, Beyotime Institute of Biotechnology) for 2 h at room temperature. Finally, the bands were assessed with BeyoECL Plus (P0018, Beyotime Institute of Biotechnology). An evaluation of target protein expression was performed using ImageJ version 1.38 (National Institutes of Health). The level of the protein of interest was normalized to the GAPDH level, which acted as the internal control in the experiments.

### Dual-luciferase reporter assay

TargetScan 7.1 (http://www.targetscan.org) was used to predict the binding sites for miR-9-5p with the 3′-UTR of StarD13. The StarD13 3′-UTR reporter plasmid (StarD13 3′-UTR WT) and StarD13 3′-UTR reporter plasmid with a mutant at the miR-9-5p-binding site (StarD13 3′-UTR MUT) were purchased from Creative Biogene. Subsequently, cells were seeded into 24-well plates and co-transfected with StarD13 3′-UTR WT or StarD13 3′-UTR MUT reporter vectors, and miR-9-5p inhibitor or NC using lipofectamine 2000 reagent (Invitrogen) for 24 h. The relative luciferase activities were analyzed after transfection for 24 h using the Dual-Luciferase Reporter Assay System (Promega) according to the manufacturer’s protocol. Firefly luciferase activities were normalized to Renilla luciferase activities.

### Statistical analysis

All results are shown as means ± SD. Statistical analyses were carried out using SPSS software version 21.0 (SPSS). Comparisons between two groups were done using Student’s *t*-test, and multiple comparisons with a one-way analysis of variance. *p* < 0.05 was considered statistically significant.

## Results

### MiR-9-5p is highly upregulated while StarD13 is downregulated in prostate cancer cells

**Fig. 1:**
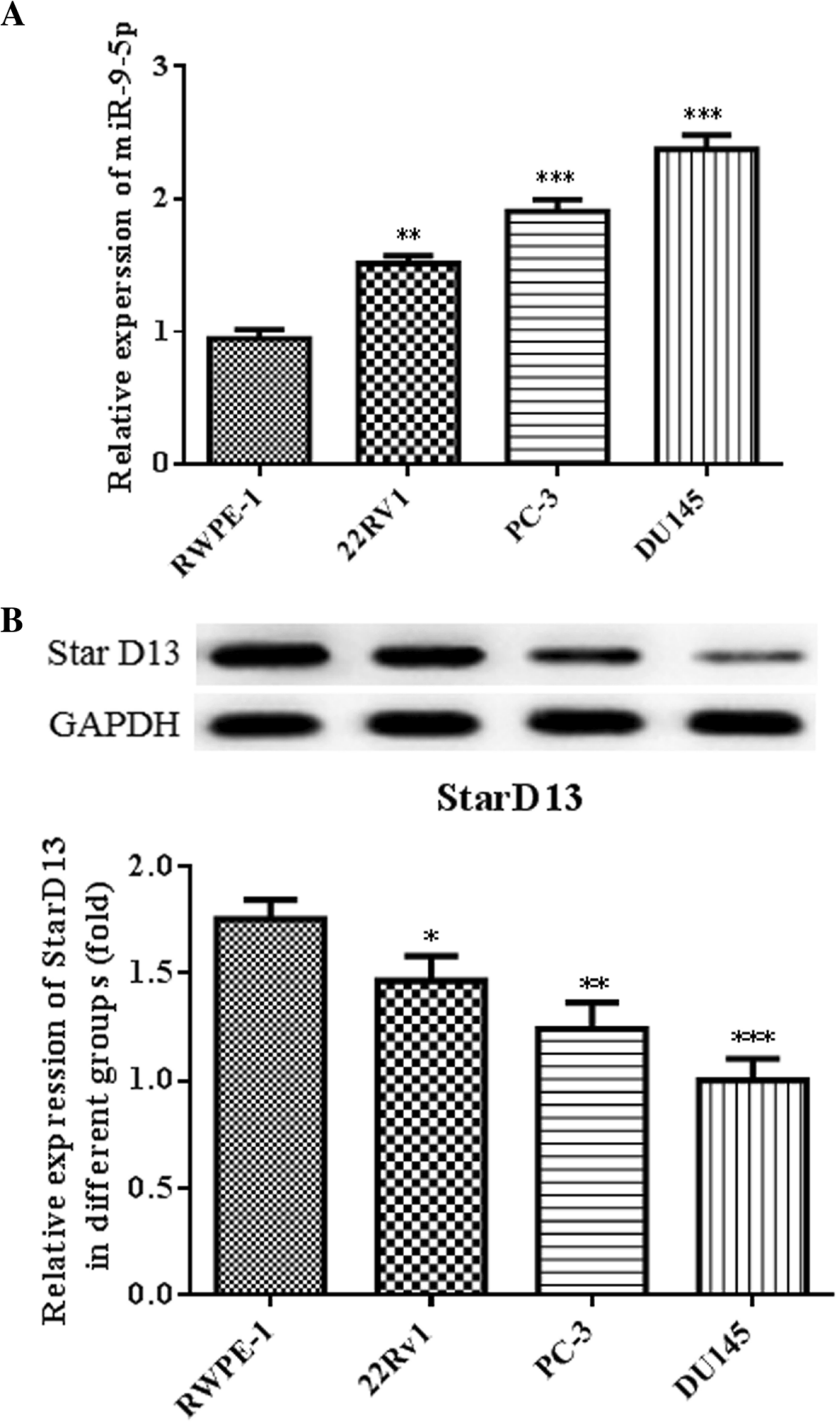
The expression levels of miR-9-5p and StarD13 in cells. **a** The mRNA expressions of miR-9-5p in RWPE-1, 22RV1, PC-3 and DU145 cells were determined using quantitative RT-PCR. **b** The protein expression levels of StarD13 in RWPE-1, 22RV1, LAPC4 and DU145 cells were assessed via western blot. *p < 0.05; **p < 0.01; ***p < 0.001 vs. RWPE-1group. All experiments were performed with at least three replicates

MiR-9-5p expression in RWPE-1, 22RV1, PC-3 and DU145 cells was assessed using real-time quantitative PCR. MiR-9-5p was more highly expressed in prostate cancer cells than normal prostate epithelial cells, especially in the DU145 and PC-3 cell lines (Fig. 1a). StarD13 expression in prostate cancer cells was notably lower, particularly in DU145 cells (Fig. 1b). The data suggest that miR-9-5p might serve as an oncogene in prostate cancer. DU145 and PC-3 cells were chosen for further experiments.

### MiR-9-5p inhibitor suppresses cell growth, invasion and migration

**Fig. 2:**
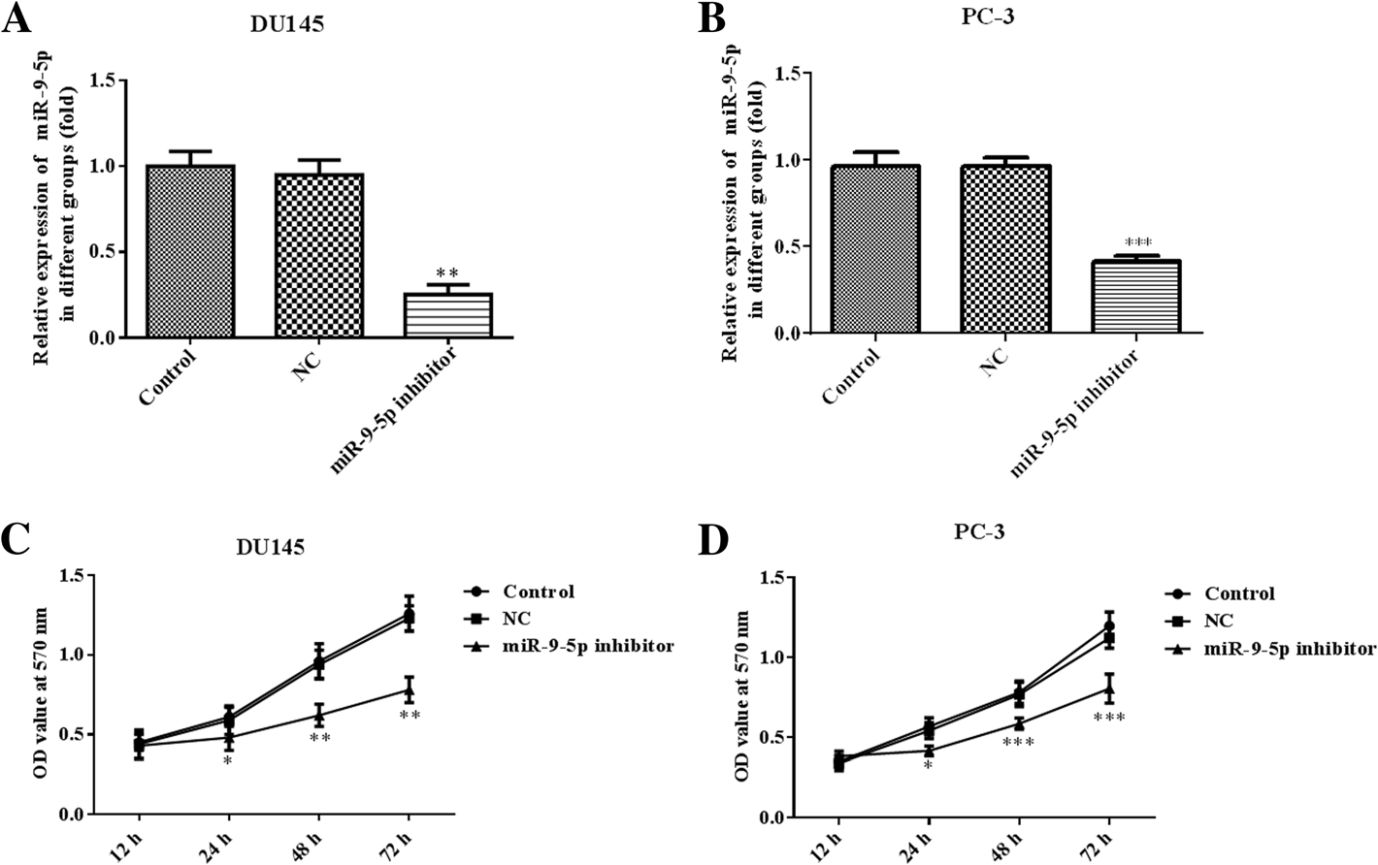
The levels of miR-9-5p expression and cell proliferation in DU145 and PC-3 cells transfected with an miR-9-5p inhibitor. The mRNA expression of miR-9-5p in DU145 cells (**a**) and PC-3 cells (**b**) transfected with miR-9-5p inhibitor or NC were determined using quantitative RT-PCR. DU145 cells (**c**) and PC-3 cells (**d**) were transfected with miR-9-5p inhibitor for 12, 24, 48 and 72 h, and cell proliferation was measured by CCK8 assay. *p < 0.05; **p < 0.01; ***p < 0.001 vs. NC group. All experiments were performed with at least three replicates

**Fig. 3:**
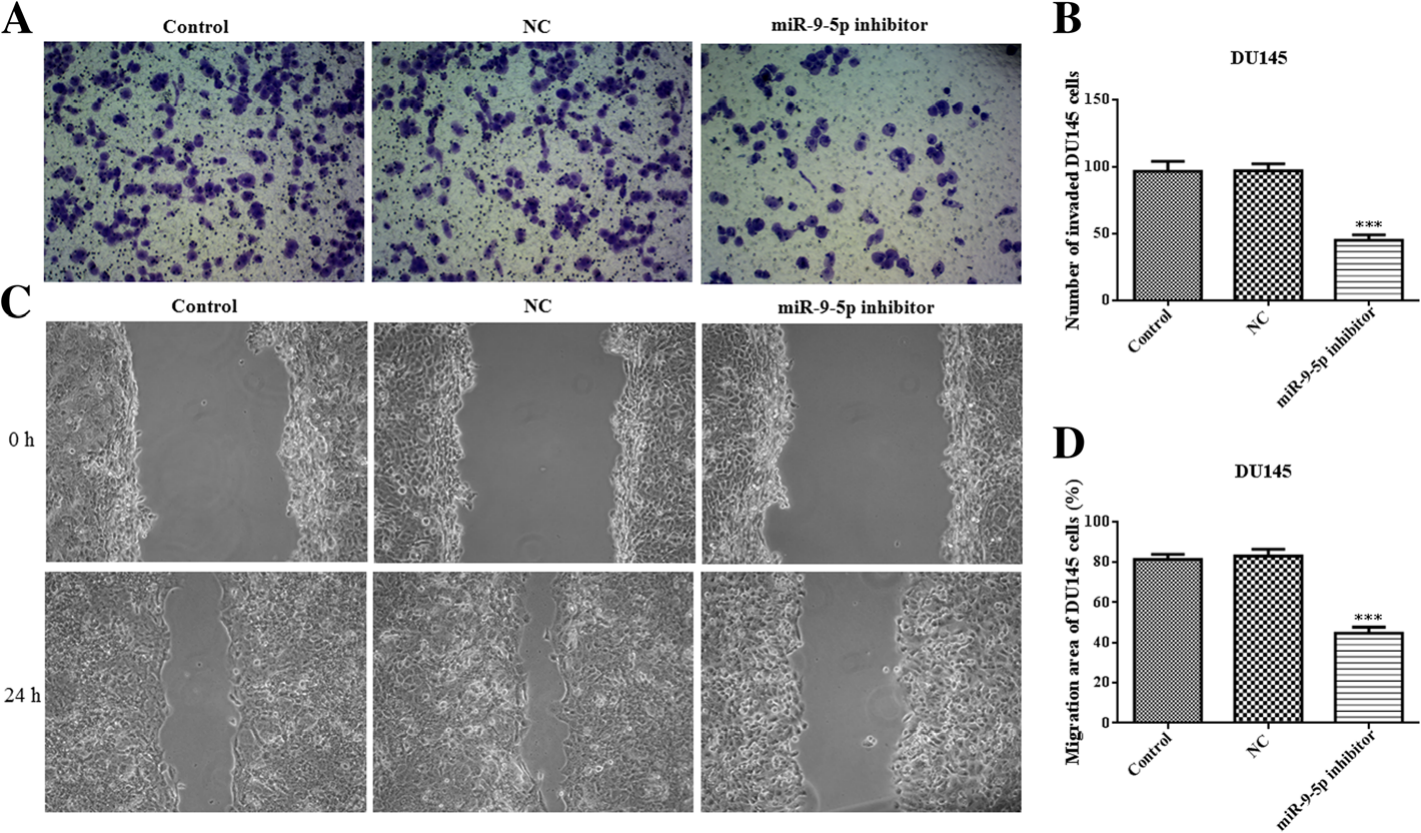
Downregulation of miR-9-5p inhibits cell invasion and migration in DU145 cells. **a** through **b** - Cell invasion of DU145 cells with an miR-9-5p inhibitor or NC transfection was evaluated using a transwell assay. **c** through **d** - U145 cells were transfected with the miR-9-5p inhibitor and cell migration was assessed using the wound-healing assay. **p < 0.01 vs. NC group; ***p < 0.001 vs. NC group. All experiments were performed with at least three replicates

**Fig. 4:**
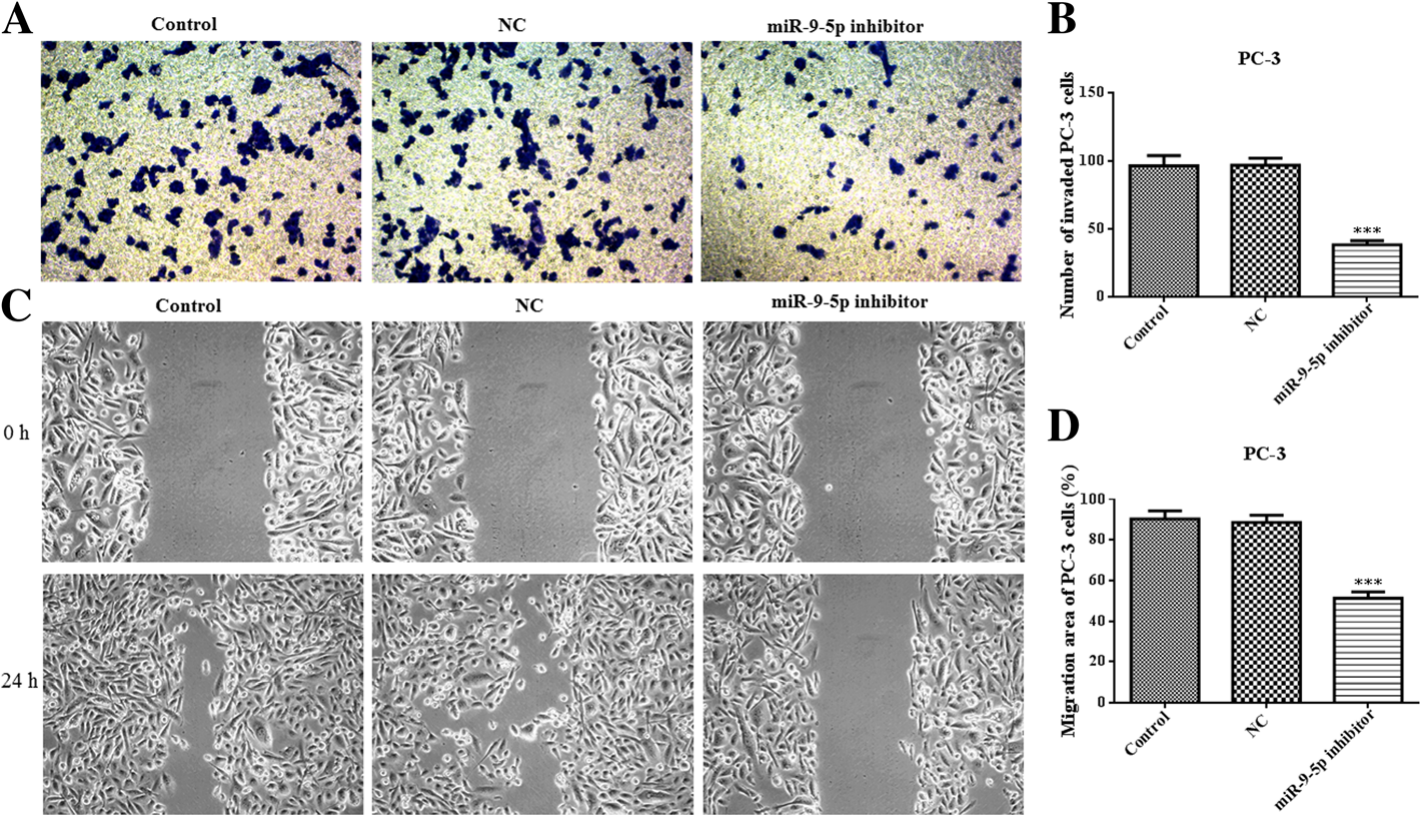
Downregulation of miR-9-5p inhibits cell invasion and migration in PC-3 cells. **a**  through **b** -Cell invasion of PC-3 cells with an miR-9-5p inhibitor or NC transfection was evaluated using a transwell assay. **c** through **d** - PC-3 cells were transfected with the miR-9-5p inhibitor and cell migration was assessed using the wound-healing assay. ***p < 0.001 vs. NC group. All experiments were performed with at least three replicates

To assess miR-9-5p effects in prostate cancer, DU145 or PC-3 cells were cultured and transfected with an miR-9-5p inhibitor. Compared with the control and negative control (NC) groups, the expression levels of miR-9-5p were significantly lower in DU145 and PC-3 cells (Fig. 2a and b). CCK8 assay showed that downregulation of miR-9-5p markedly inhibited the proliferation of DU145 and PC-3 cells (Fig. 2c and d). Moreover, the invasion assay showed that downregulation of miR-9-5p observably attenuated the invasion of DU145 cells (Fig. 3a and b). Furthermore, the migration capacity of DU145 cells after miR-9-5p downregulation was significantly suppressed, as shown with the wound-healing assay (Fig. 3c and d). The changes in invasion and migration in PC-3 cells after miR-9-5p knockdown were in accordance with the results for DU145 cells (Fig. 4).

**Fig. 5:**
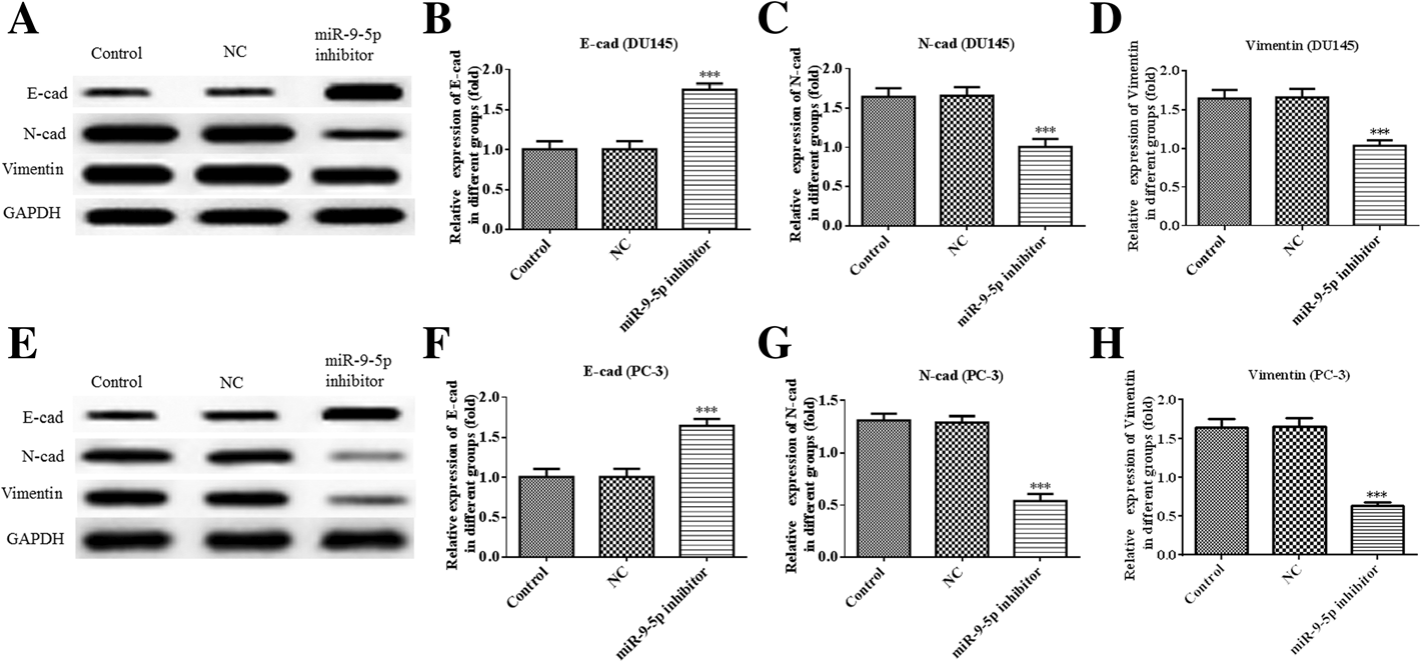
Downregulation of miR-9-5p inhibits the expression of EMT-related proteins in DU145 and PC-3 cells. **a** through **d** – The protein expressions and quantifications of E-cadherin (E-cad), N-cadherin (N-cad) and vimentin and in DU145 cells. **e** through **h** – The protein expressions and quantifications of E-cad, N-cad and vimentin and in PC-3 cells

Importantly, E-cad was upregulated while N-cad and vimentin were downregulated in both DU145 and PC-3 cells (Fig. 5). These findings demonstrate that miR-9-5p knockdown suppresses DU145 and PC-3 cell viability, invasion, migration and EMT.

### Overexpression of StarD13 inhibited cell proliferation, invasion and migration

**Fig. 6:**
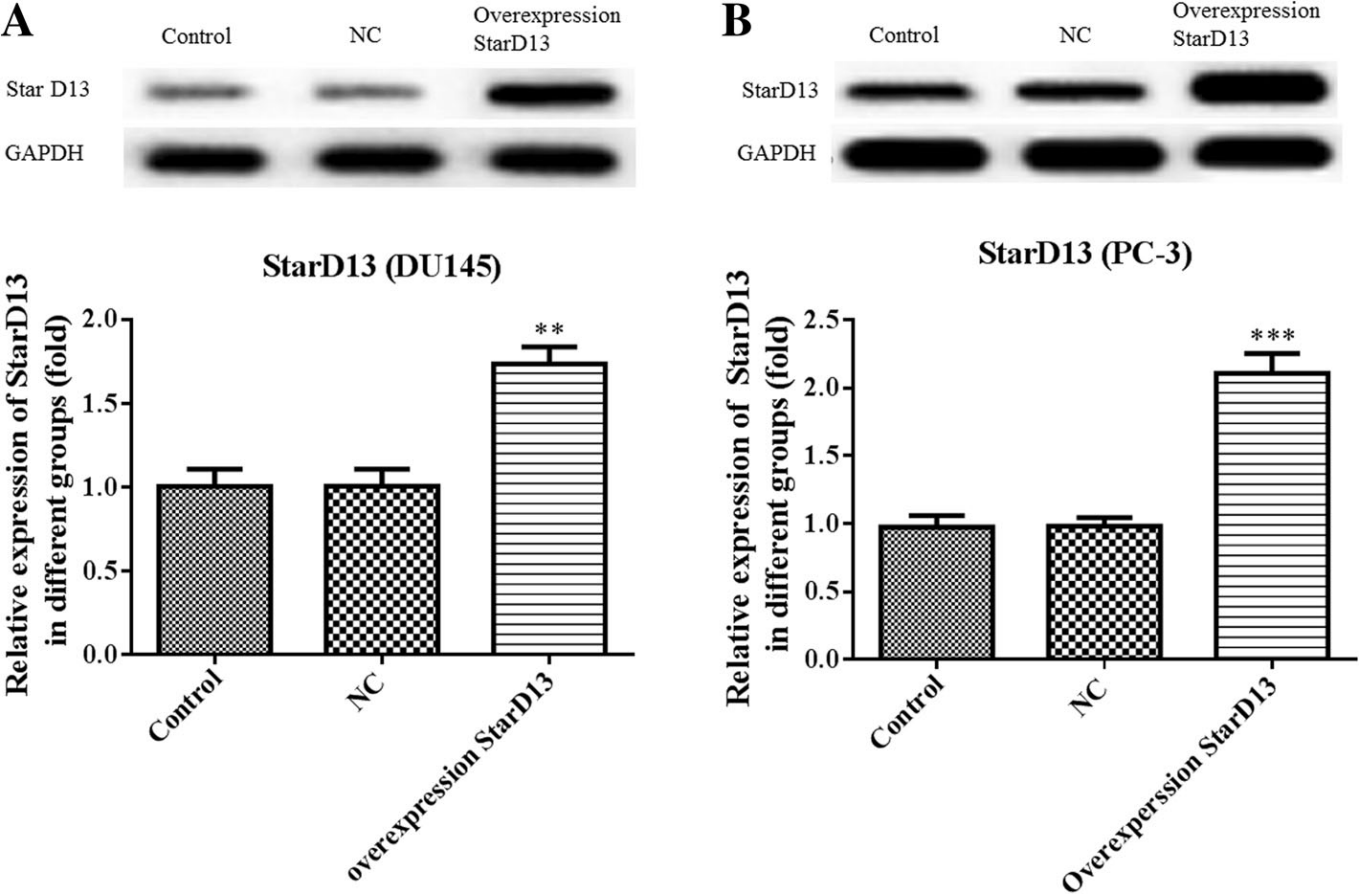
The protein expression level of StarD13 in DU145 cells and PC-3 cells transfected with pcDNA3.1 plasmid. The expression levels in DU145 cells (**a**) and PC-3 cells (**b**) were determined via western blot. **p < 0.01; ***p < 0.001 vs. NC group. All experiments were performed with at least three replicates

**Fig. 7:**
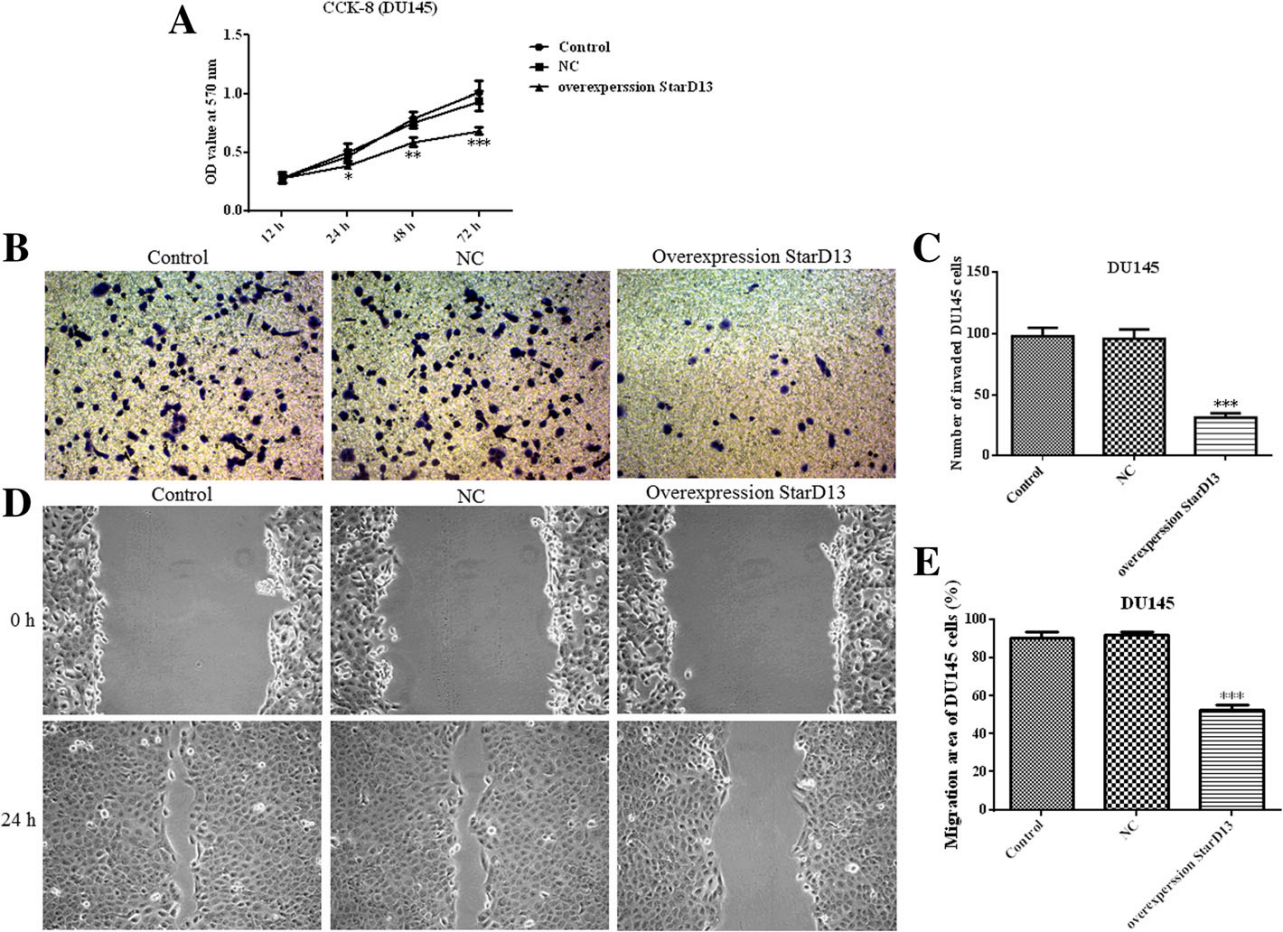
Cell proliferation, invasion and migration in DU145 cells transfected with the pcDNA3.1 plasmid of StarD13. **a** CCK8 assay results. **b** through **c** - Transwell invasion assay results. **d** through **e** - Wound-healing assay results. **p < 0.01; ***p < 0.001 vs. NC group. All experiments were performed with at least three replicates

**Fig. 8:**
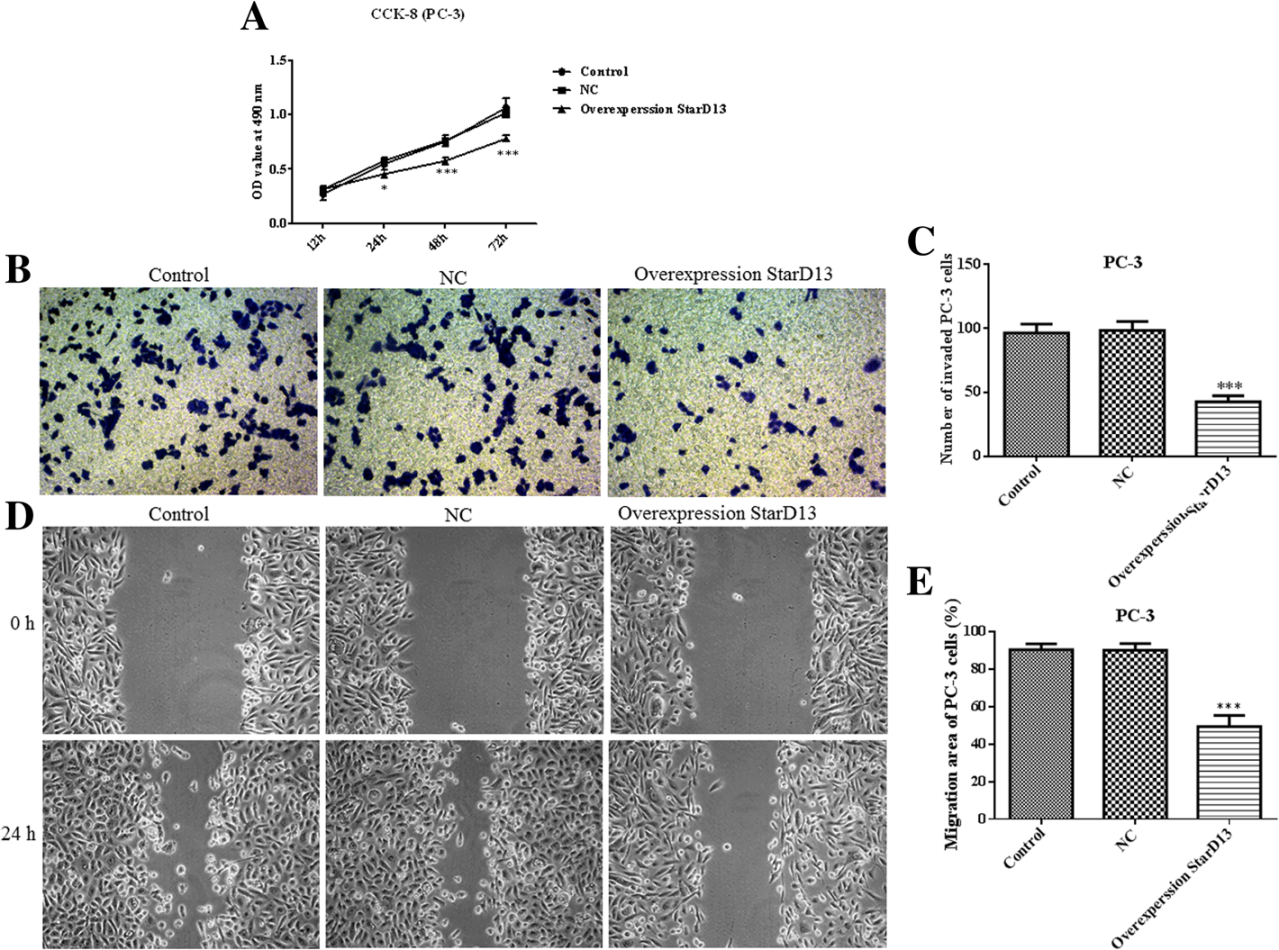
Cell proliferation, invasion and migration in PC-3 cells transfected with the pcDNA3.1 plasmid of StarD13. **a** CCK8 assay results. **b** through **c** - Transwell invasion  assay results. **d** through **e** - Wound-healing assay results. **p < 0.05; ***p < 0.001 vs. NC group. All experiments were performed with at least three replicates

StarD13 overexpression in DU145 and PC-3 cells was used to verify its impact on proliferation, invasion and migration. The results for non-overexpressing cells show that StarD13 is normally significantly downregulated in the two cell lines (Fig. 6a and b). When it is overexpressed, the capacity of DU145 cell proliferation (Fig. 7a), invasion (Fig. 7b and c) and migration (Fig. 7d and e) was suppressed significantly. All the results for PC-3 cells were in line with those for DU145 cells (Fig. 8). Collectively, these data indicate that StarD13 plays a vital role in cell growth, invasion and migration.

### StarD13 is a direct target of miR-9-5p

**Fig. 9:**
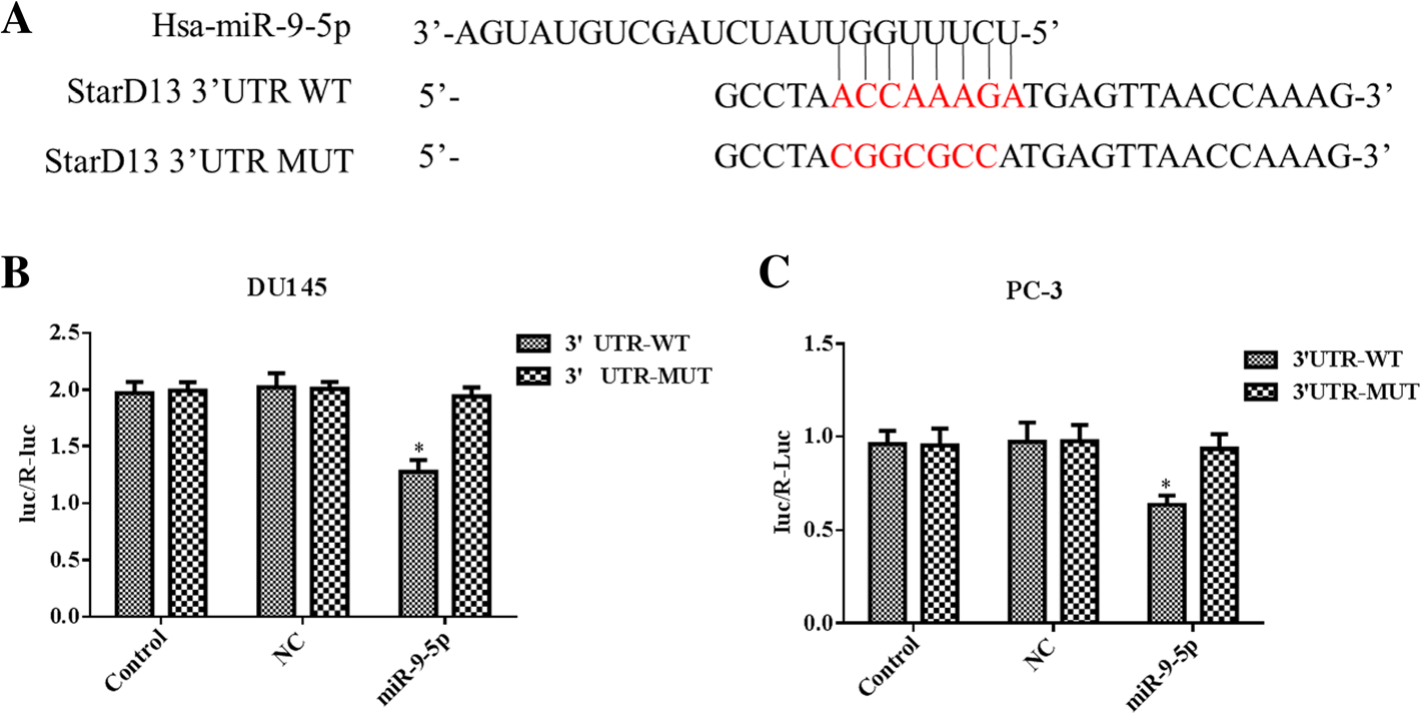
StarD13 is a direct target of miR-9-5p. **a** The 3′-UTR of StarD13 mRNA was found to contain the complementary sequence of miR-9-5p. **b** and **c** Downregulated miR-9-5p expression significantly suppressed the dual-luciferase activity of the wild-type (WT) 3′-UTR of StarD13 but not the mutant (MUT) 3′-UTR of StarD13 in DU145 cells (**b**) or PC-3 cells (**c**). *p < 0.05 vs. 3′-UTR MUT. All experiments were performed with at least three replicates

Our biomatics analysis predicted that miR-9-5p could target the 3′-UTR of StarD13. The dual-luciferase reporter assay was performed to confirm whether miR-9-5p can bind to the 3′-UTR of StarD13 (Fig. 9a). The results suggest that the miR-9-5p inhibitor attenuated the luciferase activity of wild-type (WT) StarD13 3′-UTR in DU145 (Fig. 9b) and PC-3 (Fig. 9c) cells.

**Fig. 10:**
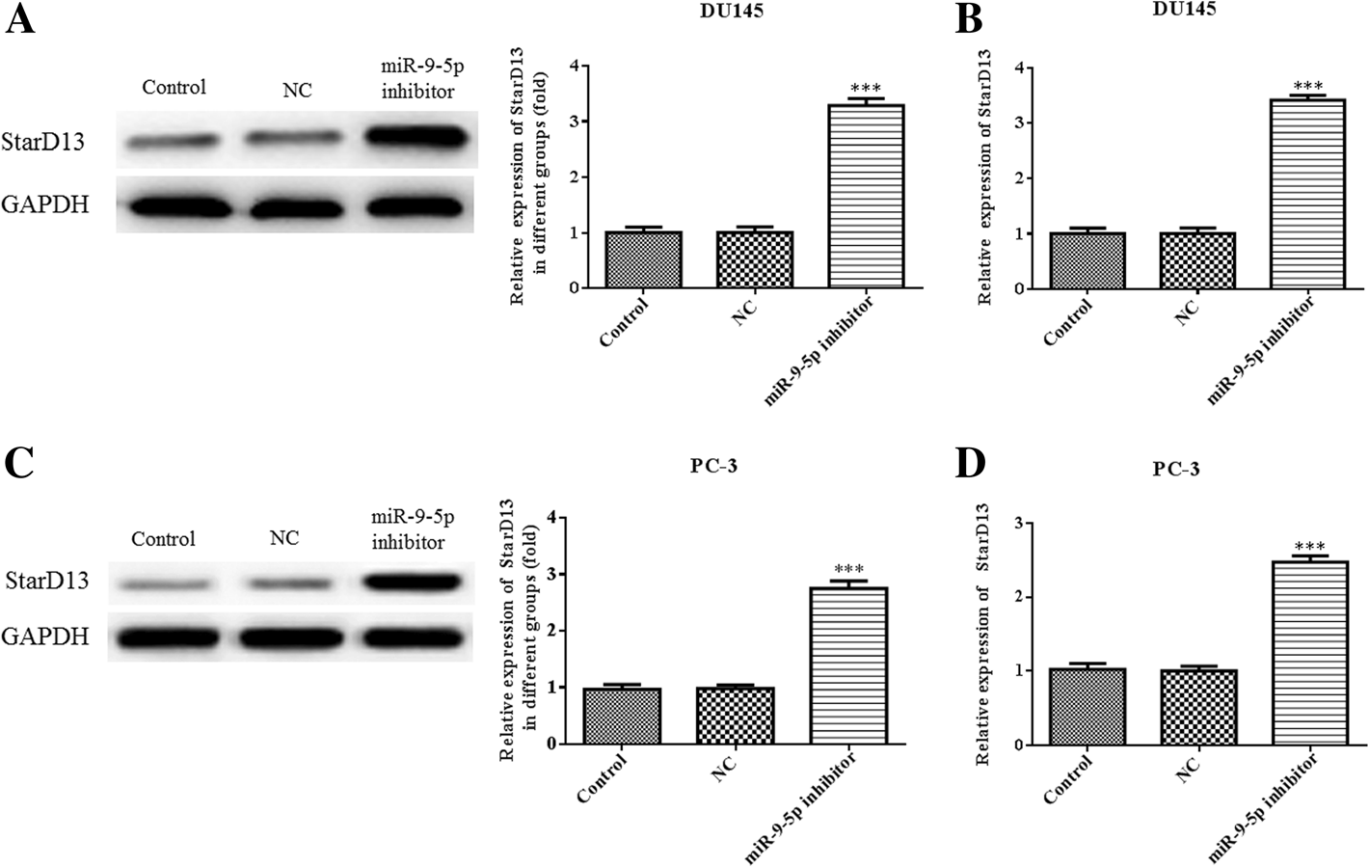
The expression levels of StarD13 in DU145 and PC-3 cells transfected with an miR-9-5p inhibitor. The protein (**a**) and mRNA (**b**) expression levels of StarD13 in DU145 cells with miR-9-5p inhibitor or NC transfection were determined, respectively using quantitative RT-PCR or western blot. The protein (**c**) and mRNA (**d**) expression levels of StarD13 in PC-3 cells with miR-9-5p inhibitor or NC transfection were determined, respectively using quantitative RT-PCR or western blot. ***p < 0.001 vs. NC group. All experiments were performed with at least three replicates

To further verify that StarD13 was a direct target of miR-9-5p, StarD13 mRNA and protein expression levels were measured using quantitative real-time PCR and western blot, respectively. The miR-9-5p inhibitor increased the StarD13 mRNA and protein expression levels in DU145 (Fig. 10a and b) and PC-3 cells (Fig. 10c and d). These data all indicate that StarD13 serves as a direct target of miR-9-5p.

## Discussion

In Asia, prostate cancer seems to be gradually becoming a more serious medical and socio-economic problem, in line with what is seen in western countries [3]. MiRNAs play a critical role in the progression of many cancers, and it has been well documented that miR-9-5p can regulate the progress of tuberous sclerosis complex angiomyolipoma [21], non-small cell lung cancer [9] and breast cancer [22]. However, the effect of miR-9-5p in prostate cancer has yet to be elucidated. Our quantitative real-time PCR results showed that miR-9-5p was highly expressed in prostate cancer. The data demonstrate that miR-9-5p may be an oncogene for prostate cancer.

To further explore the effects of miR-9-5p in prostate cancer, an miR-9-5p inhibitor was transfected into DU145 or PC-3 cells. The results of the CCK-8 assay showed that downregulation of miR-9-5p repressed proliferation of DU145 and PC-3 cells. Since tumor metastasis serves as a leading mortality factor in prostate cancer patients, we performed transwell and wound-healing assays to determine the migration and invasion ability in the prostate epithelial cell line. We found that miR-9-5p silencing suppressed the invasion and migration of DU145 and PC-3 cells. EMT has also been deemed vital in the regulation of the initial phase of prostate cancer metastasis. Its significance in mediating aggressiveness in the progression of prostate cancer has drawn increasing attention in recent years [23]. A huge body of evidence has demonstrated that EMT is not only involved in cell migration and invasion but also in chemo-resistance or radio-resistance in different tumor types [24]. Our findings confirmed miR-9-5p as a pivotal regulator of EMT in DU145 and PC-3 cells, which could be recognized from the increase in E-cad expression accompanied by a decrease in N-cad and vimentin expression. These findings confirmed that miR-9-5p might exert a significant role in the progress of prostate cancer.

STAR-related lipid transfer domain containing 13 (StarD13), also known as deleted in liver cancer 2 protein (DLC-2), may be involved in the regulation of cell proliferation and apoptosis, acting as a tumor suppressor in hepatocellular carcinoma [16, 25]. In this study, proliferation, invasion and migration were reduced in StarD13-overexpressing DU145 and PC-3 cells. StarD13 is a well-documented direct and functional target of miR-125b in gastric cancer, with its upregulation inhibiting invasion and metastasis [18]. However, the effect of StarD13 on cell migration remains controversial. Previous studies in breast cancer lines have shown that knockdown of StarD13 did not affect the migratory behavior of cells and the regulatory effect was performed by DCL-1 [26]. However, recent evidence supports StarD13 being a tumor suppressor in breast cancer and that it regulates cell motility and invasion [27]. The StarD13 gene as a target of miR-720 could regulate the abilities of cell growth, migration and invasion in colorectal cancer [28]. Earlier in vivo mice experiments showed that StarD13 was a metastasis suppressor gene, which is consistent with our results [29].

StarD13 was predicted to be a direct target of miR-9-5p in prostate cancer in our bioinformatics analysis. This prediction was validated using the dual-luciferase reporter assay. Silencing of miR-9-5p upregulated StarD13 mRNA and protein, confirming our results.

## Conclusions

The results of this study show that miR-9-5p exerts considerable effects on proliferation, invasion and migration in cells of the prostate cancer lines DU145 and PC-3. This effect occurs via targeting of StarD13, indicating that miR-9-5p may act as a tumor suppressor and suggesting an effective therapeutic strategy in prostate cancer.

## Abbreviations

miRNA: microRNA
MUT: Mutant type
NC: Negative controls
StarD13: StAR-related lipid transfer domain containing 13
UTR: Untranslated region
WT: Wild type
